# Exploring the genetic causal association of TIMP3 on CKD and kidney function: a two-sample mendelian randomization

**DOI:** 10.3389/fgene.2024.1367399

**Published:** 2024-05-07

**Authors:** Huang Chen, Lixun Chen, Yufeng Chen, Qinyu Guo, Shirong Lin

**Affiliations:** ^1^ Department of Emergency, Fujian Provincial Hospital, Fuzhou, Fujian, China; ^2^ Shengli Clinical Medical College, Fujian Medical University, Fuzhou, Fujian, China; ^3^ Fujian Provincial Key Laboratory of Emergency Medicine, Fujian Provincial Hospital, Fuzhou, Fujian, China; ^4^ Fujian Provincial Institute of Emergency Medicine, Fujian Provincial Hospital, Fuzhou, Fujian, China; ^5^ Fujian Emergency Medical Center, Fujian Provincial Hospital, Fuzhou, Fujian, China

**Keywords:** mendelian randomization, GWAS, TIMP3, CKD, kidney function

## Abstract

**Background:** Numerous studies have demonstrated a positive association between the level of tissue inhibitor of metalloproteinase 3 (TIMP3) and chronic kidney disease (CKD). Nevertheless, whether those associations reflect causal links still to be determined. This study intended to research the causal relationship of TIMP3 with CKD and markers of kidney function, such as creatinine‐based estimated glomerular filtration rate (eGFRcrea), cystatin C‐based estimated glomerular filtration rate (eGFRcys), eGFRcrea in diabetics (eGFRcrea (DM)) and eGFRcrea in non diabetics (eGFRcrea (No DM)).

**Methods:** In this study, we investigated the causal relationships between TIMP3 and CKD and kidney function markers using a two-sample Mendelian randomization (MR) technique. We used summary level datasets for TIMP3 and CKD from genome-wide association studies that we were able to access through the study by Suhre K and Pattaro C.

**Results:** We found that TIMP3 had a significant positive causal effect on the risk of CKD (Inverse variance weighted (IVW):odds ratio (OR):0.962, 95% confidence interval (CI): (0.936-0.988),P:0.005). However TIMP3 levels had no significant effect on risk of eGFRcys (PIVW: 0.114),eGFRcrea (PIVW:0.333). After grouping patients based on their diabetes status, we found that genetically higher levels of TIMP3 had a significant impact on eGFRcrea in participants without diabetes (OR:1.003,95%CI (1.001-1.006),P IVW:0.007), but not in participants with diabetes (PIVW = 0.057). Heterogeneity and pleiotropy analyses were carried out to verify the accuracy of the MR findings. Their findings were all not statistically significant.

**Conclusion:** Our study suggests that TIMP3 may be causally associated with CKD and eGFRcrea (No DM)in people of European ancestry. Strategies aimed to increase TIMP3 levels may provide new ways to delay the deterioration of renal function.

## 1 Introduction

Recently, chronic kidney disease (CKD) has drawn increasing interest in the field of human health (Shi et al., 2018). The treatment for CKD often imposes a significant finacial burden on both society and families (Xie et al., 2018). Renal function test markers serve as indicators to evaluate function of the kidney. Indicators of kidney function that are most frequently utilized include estimated glomerular filtration rate (eGFR). It comprises two components: eGFRcrea, which is based on creatinine, and eGFRcys, which is based on cystatin C (Gowda et al., 2010; Dahlén and Björkhem-Bergman, 2022). Then, depending on whether the patient had diabetes or not, the eGFRcrea was divided into two types in our study:eGFRcrea in diabetics (eGFRcrea (DM)) and eGFRcrea in non-diabetics (eGFRcrea (No DM)).

Tissue inhibitors of metalloproteinases (TIMPs) are endogenous protein regulators who modulate the activity of matrix metalloproteinases (MMPs) (Fan and Kassiri, 2020). TIMP3 suppresses all MMPs and possesses the broadest inhibitory scope against metalloproteinases (Brew and Nagase, 2010). Furthermore, TIMP3 expression in the kidney is higher when compared to other TIMPs (Catania et al., 2007). According to earlier research, TIMP3 is essential for the development of chronic kidney illnesses, including renal carcinoma, diabetic nephropathy, chronic nephritis, and other kidney diseases (Kassiri et al., 2009; Fiorentino et al., 2013; Liu and Zhang, 2021; Zhou et al., 2022). However, no randomized controlled trials (RCTs) have been carried out to investigate the connection between CKD and TIMP3.

Mendelian randomization (MR) is a method of epidemiological research that has undergone significant developments in the last few years (Bowden and Holmes, 2019). Genetic variation is used as an instrumental variable (IV) for the estimation of the causal relationship between exposure and outcome (Emdin et al., 2017). The advantage of MR is that it creates a scenario similar to randomized controlled trial, which effectively minimized the influence of residual confounders and reverse causality (Davey Smith and Hemani, 2014). In light of this, the purpose of our work was to use a two-sample MR technique to examine the relationship between TIMP3, CKD and renal function (measured by biochemical markers and the presence or absence of diabetes). Two large-sample genome-wide association studies (GWAS) of European ancestry provided summary statistics that we used.

## 2 Materials and methods

**FIGURE 1:**
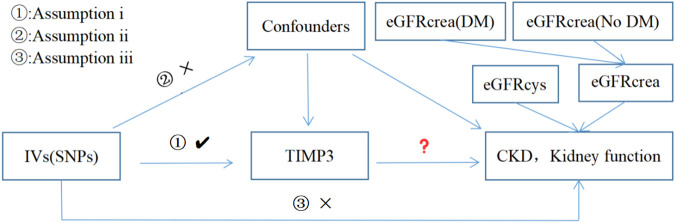
Three assumptions for IVs in MR analysis. Abbreviations: TIMP3, tissue inhibitor of metalloproteinase 3; SNPs, single nucleotide polymorphisms; IVs, instrumental variables; CKD, chronic kidney disease; eGFRcrea, estimated glomerular filtration rate; eGFRcys, cystatin C estimated glomerular filtration rate; DM, diabetes mellitus.

Figure 1 provides a concise view of the two-sample MR design between TIMP3 and CKD, as well as kidney function. We performed MR analyses using summary statistics from a GWAS to investigate the impact of TIMP3 on the risk of CKD and kidney function. In order to ensure the validity of IVs, Assumptions needed to be met in three key areas (Davies et al., 2018): (i)Exposure should be significantly influenced by genetic variants; (ii)it is imperative that the genetic variants employed are free from any confounding factors; (iii)It is only the exposure indirectly that affects the outcome, and not other biological pathways.

### 2.1 Data sources

The TIMP3 summary-level data used in this investigation was obtained from a cohort study of approximately 1,000 individuals, which was made available through the Suhre K study (Suhre et al., 2017). All summary statistics pertaining to CKD and kidney function were obtained from the study of Pattaro C (Pattaro et al., 2016) which exclusively used continuous biomarkers, including CKD (N = 117165), eGFRcys (N = 32834), eGFRcrea (N = 133413),eGFRcrea (No DM) (N = 118448), and eGFRcrea (DM) (N = 11522). This study’s data are all of European descent. Moreover, the open-access GWAS dataset at https://gwas.mrcieu.ac.uk/contains the summary data for both GWAS studies. Further details regarding each dataset can be found in Table 1.

**TABLE 1:** Source and sample size of GWAS summary statistics.

Abbreviation	PMID (Data source)	Sample size	Population	GWAS ID
TIMP3	28240269	1,000	European	prot-c-2480_58_3
CKD	26831200	117,165	European	ebi-a-GCST003374
EGFRcrea	26831200	133,413	European	ebi-a-GCST003372
eGFRcrea (DM)	26831200	11,522	European	ebi-a-GCST003373
eGFRcrea (No DM)	26831200	118,448	European	ebi-a-GCST003401
EGFRcys	26831200	32,834	European	ebi-a-GCST003375

Abbreviation:GWAS ID, identification of genome-wide association studies; TIMP3, Tissue inhibitor of metalloproteinase 3; CKD, chronic kidney disease; eGFRcrea, estimated glomerular filtration rate; eGFRcys, cystatin C estimated glomerular filtration rate; DM, diabetes mellitus.

### 2.2 IVs selection

We will use the following criteria as SNP screening for TIMP3 in order to ensure the effectiveness of IVs.First, the genome-wide significance was set at p < 5e-8. Then, in order to mitigate the impact of linkage imbalance, we conducted the caking process (r2:0.1; window:10,000 KB) (Purcell et al., 2007) reference 1,000 genomes European Panel (Auton et al., 2015). We ended up with seven SNPs in Table 2. When reconciling exposure and outcome data, palindromic SNPS with moderate allele frequencies were removed. A MR Steiger filter test was then performed and SNPs suggesting reverse causality were removed (Hemani et al., 2017). Furthermore, PhenoScanner V2 was searched for phenotypes linked to these SNPs (Kamat et al., 2019). Here, we discovered that there was no significant correlation between any of these IVs and possible confounders (risk factors for renal impairment). And ensure that the F statistic is > 10, excluding the possibility of weak instrumental variable bias (Burgess and Thompson, 2011).

**TABLE 2:** Summary information on the instruments variable of exposure (TIMP3).

SNP	chr	Samplesize	Eaf	Beta	se	Pval	F Statistics
rs3788495	22	996	0.4284	0.2633	0.0432	1.53E-09	37.1231
rs135150	22	997	0.8469	−0.5332	0.0603	4.38E-18	77.9535
rs241890	22	994	0.1665	−0.4995	0.0529	2.65E-20	88.8776
rs5754249	22	997	0.5474	−0.3203	0.0429	1.71E-13	55.7487
rs34843069	22	993	0.0444	−0.7453	0.1104	2.52E-11	45.4706
rs2097326	22	992	0.728	−0.9307	0.0397	4.42E-97	549.2615
rs5754266	22	993	0.1494	−0.4177	0.0555	1.16E-13	56.5566

SNP, single nucleotide polymorphism; Chr, chromosome, eaf:effect allele frequency; bate, beta.exposure, se:standard error; *p*-value, the value for the genetic association.

### 2.3 MR analysis

Examined the genetic association between IVs of TIMP3 level and the risk of CKD using a two-sample MR analysis. We used inverse variance weighting (IVW) (Pierce et al., 2011) as the main analysis method, MR-Egger, weighted median (WM), weighted model, and simple model as supplementary methods (Bowden et al., 2015; Bowden et al., 2016; Hartwig et al., 2017; Hemani et al., 2018). Then, we evaluated the second and third hypotheses indirectly by performing a sensitivity analysis. First, we used the Cochrans Q test to determine the inter-IV heterogeneity (Cohen et al., 2015). Secondly, the MR-PRESSO global test (Verbanck et al., 2018) and MR-Egger regression (Bowden et al., 2015) were used to test the horizontal pleiotropy of IVs. Finally, a leave-out analysis was conducted to determine the potential bias effect of a single SNP on MR Estimates (Burgess et al., 2017). We conducted MR Analysis using the TwoSampleMR R package (version 0.5.6) in RStudio software (version 4.3.1). We consider *p* < 0.05 as statistically significant.

## 3 Result

**FIGURE 2:**
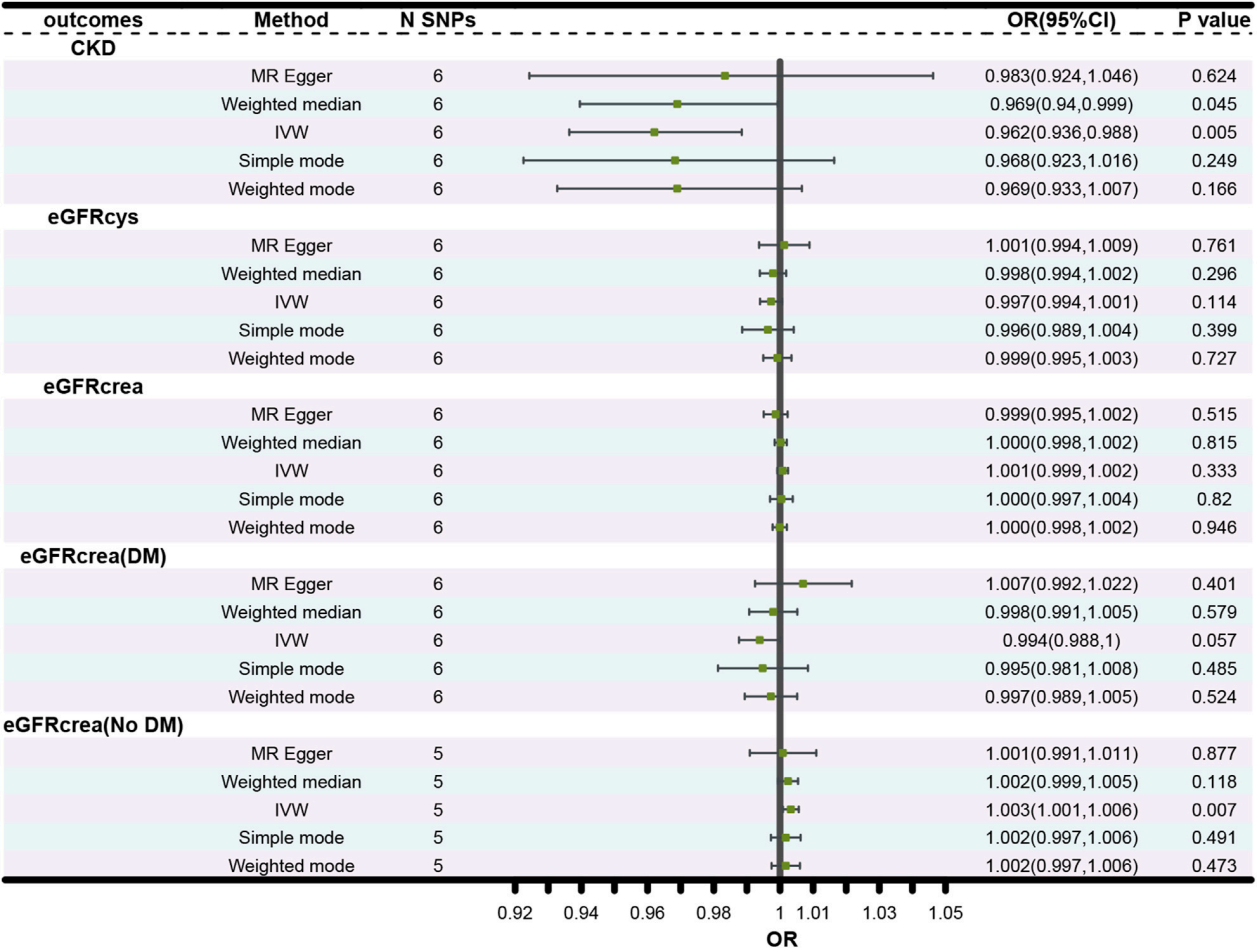
Forest plot of the main MR study investigating the causal effect of TIMP3 on CKD and kidney function. Abbreviations: OR, odds ratio; CI, confidence interval; IVW, Inverse variance weighted; N SNPs, number of single nucleotide polymorphisms; CKD, chronic kidney disease; eGFRcrea, estimated glomerular filtration rate; eGFRcys, cystatin C estimated glomerular filtration rate; DM, diabetes mellitus.

**FIGURE 3:**
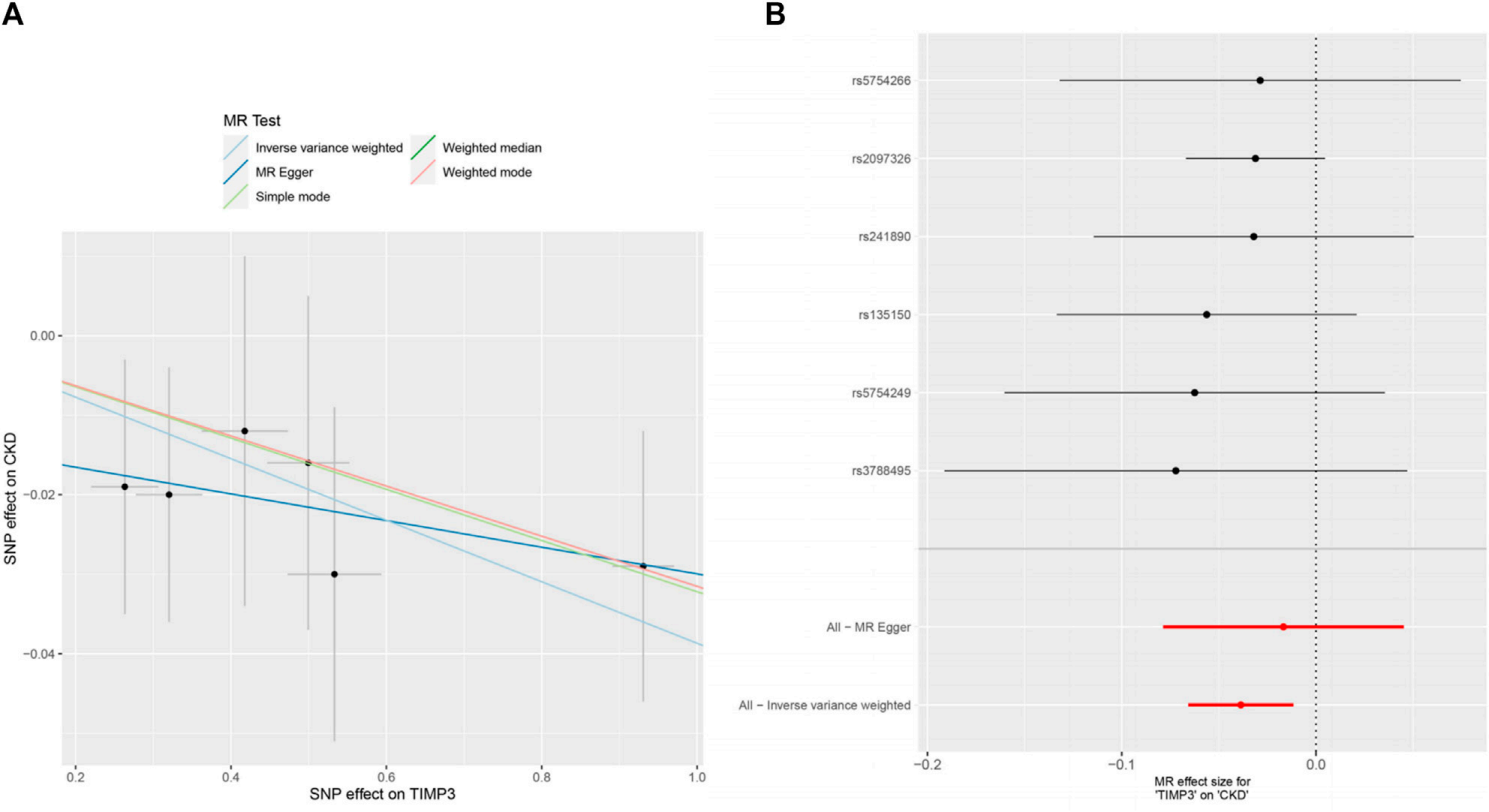
MR analysis visualizations of the effect of TIMP3 on CKD. **(A)** Scatter plot of the effect size for each SNP on TIMP3 and CKD, **(B)** Forest plotof the effect size for each SNP on TIMP3 and CKD. Abbreviations:CKD:chronic kidney disease, TIMP3:tissue inhibitor of metalloproteinase 3,SNP:single nucleotide polymorphism.

**FIGURE 4:**
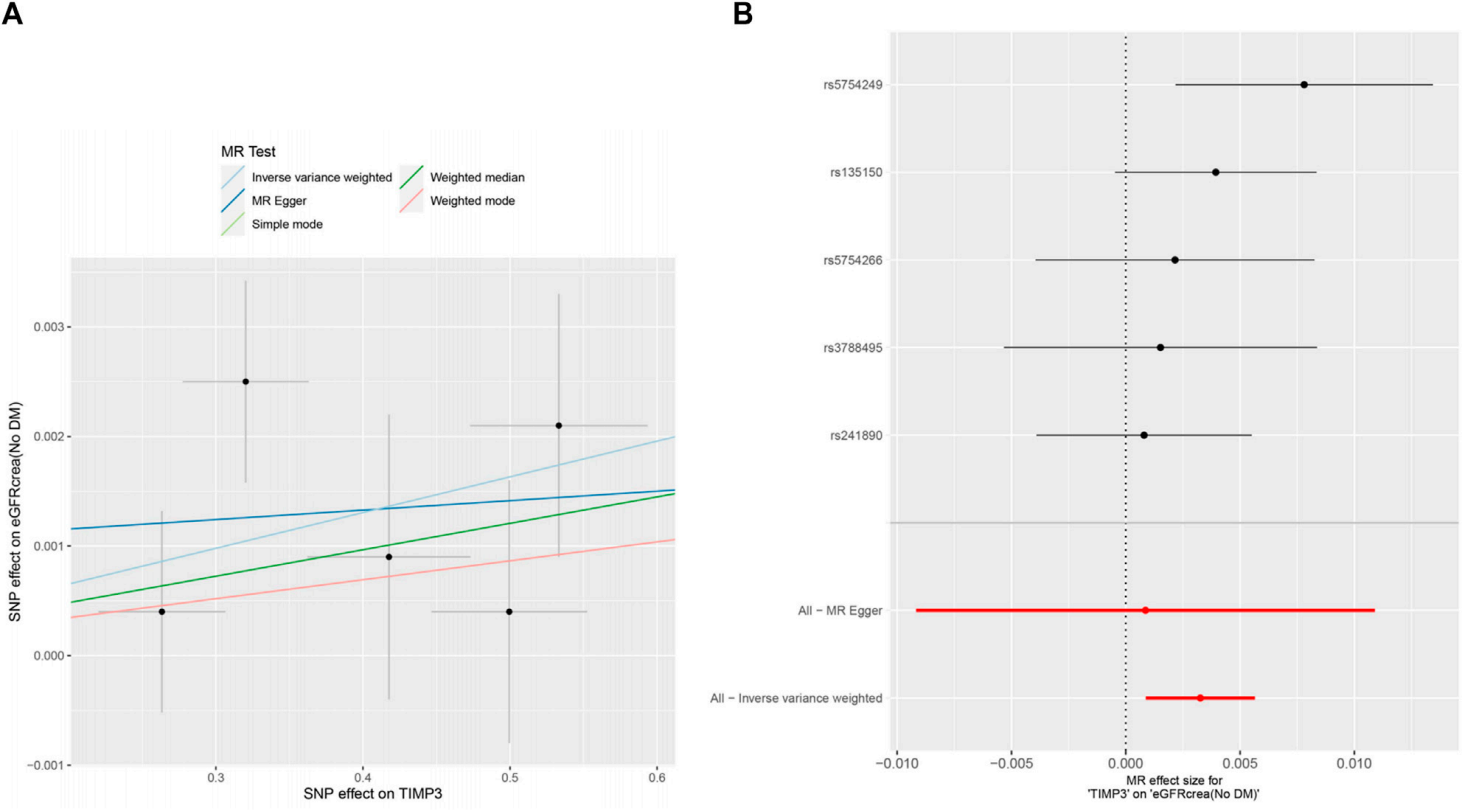
MR analysis visualizations of the effect of TIMP3 on eGFRcrea (No DM). **(A)** Scatter plot of the effect size for each SNP on TIMP3 and eGFRcrea (No DM). **(B)** Forest plotof the effect size for each SNP on TIMP3 and eGFRcrea (No DM). Abbreviations:eGFRcrea (No DM), creatinine‐based estimated glomerular filtration rate in non-diabetics; TIMP3, tissue inhibitor of metalloproteinase 3,SNP:single nucleotide polymorphism.

The forest plots (Figure 2) showed the causal effect of TIMP3 on kidney function. The IVW analysis revealed that TIMP3 has a causal impact on the probability of developing CKD (IVW: OR = 0.962, 95% CI(0.936-0.988), *p* = 0.005; WM: OR = 0.969, 95% CI(0.94-0.999), *p* = 0.045). Furthermore, we found a causal relationship between TIMP3 and eGFRcream (No DM) (IVW:OR = 1.003,95%CI (1.001-1.006),*p* = 0.007). Results of the forest and scatter plots for each outcome were shown in Figures 3, 4.

**FIGURE 5:**
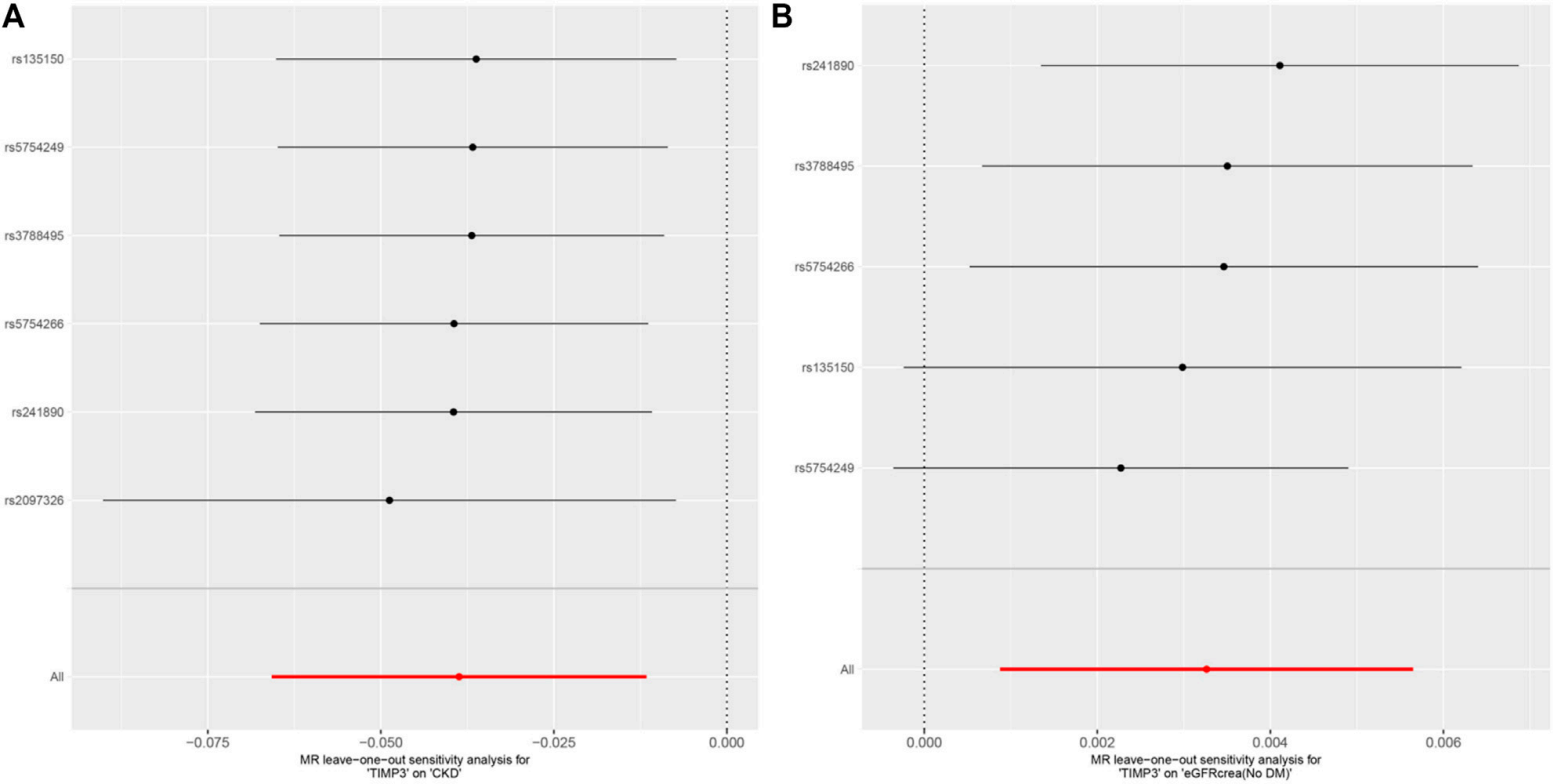
MR analysis visualizations of the effect of TIMP3 on CKD and eGFRcrea (No DM). **(A)** Scatter plot of the effect size for each SNP on TIMP3 and eGFRcrea (No DM). **(B)** Forest plotof the effect size for each SNP on TIMP3 and eGFRcrea (No DM). Abbreviations:CKD, chronic kidney disease; eGFRcrea (No DM), creatinine‐based estimated glomerular filtration rate in non-diabetics; TIMP3, tissue inhibitor of metalloproteinase 3; SNP, single nucleotide polymorphism.

Subsequently,a sensitivity analysis was then performed to evaluate how reliable our findings were. Initially, all *p* values above 0.05 in the Cochran’s Q test results showed no discernible heterogeneity among the independent variables (Table 3). Secondly, there was no indication of pleiotropy for either CKD or eGFR, as shown by the MR-PRESSO global test and MR-Egger regression with all *p* > 0.05 (Table 3). Finally, the leave-one analysis revealed that the outcomes were not influenced by any SNPs(Figure 5).

**TABLE 3:** Pleiotropy and heterogeneity tests of Mendelian randomization analyses.

Outcomes	Heterogeneity (Cochran’s Q test)			Pleiotropy		
	Method	Q test	*p*-value	Method	Effect size	*p*-value
CKD	MR Egger	0.365	0.985	MR-Egger (egger_intercept)	−0.013	0.484
	IVW	0.959	0.966	MR-PRESSO (RSSobs)*	1.861	0.947
eGFRcys	MR Egger	2.266	0.687	MR-Egger (egger_intercept)	−0.002	0.319
	IVW	3.558	0.615	MR-PRESSO (RSSobs)*	8.282	0.482
eGFRcrea	MR Egger	3.629	0.458	MR-Egger (egger_intercept)	0.001	0.272
	IVW	5.248	0.386	MR-PRESSO (RSSobs)*	10.691	0.389
eGFRcrea (DM)	MR Egger	0.885	0.927	MR-Egger (egger_intercept)	−0.008	0.121
	IVW	4.744	0.448	MR-PRESSO (RSSobs)*	9.552	0.445
eGFRcrea (No DM)	MR Egger	3.724	0.293	MR-Egger (egger_intercept)	0.001	0.660
	IVW	4.017	0.404	MR-PRESSO (RSSobs)*	6.286	0.459

IVW, inverse variance weighted; CKD, chronic kidney disease; eGFRcrea, estimated glomerular filtration rate; eGFRcys, cystatin C estimated glomerular filtration rate; DM, diabetes mellitus; MR-PRESSO, Mendelian Randomization Pleiotropy RESidual Sum and Outlier; *, No outlier were identified.

There was no causal association between TIMP3 and the other types of eGFR. The *p* values for all 3 MR methods were greater than 0.05 (Figure 2).

## 4 Discussion

In this comprehensive two-sample MR analysis of the associations between TIMP3 and kidney function, we have provided the first suggestive evidence that higher TIMP3 levels decrease CKD, and we observed a significant negative causal relationship between TIMP3 and eGFRcrea (No DM). However, we did not find any genetic associations between TIMP3 and the other kidney function biomarkers.

Dysregulation of MMPs in CKD has been well-established. Studies using mouse models have indicated that genetic targeting of selected MMPs may have detremental effects on kidney function (Wozniak et al., 2021). Numerous studies have demonstrated that MMPs may cause rupture of the glomerular basement membrane, digestion of ECM components that are linked to renal scarring and fibrosis (Zakiyanov et al., 2019), as well as the activation of inflammatory cells and induction of autophagy in kidney tissue (Toba and Lindsey, 2019; Zheng et al., 2022). However, TIMP3, an inhibitor of MMPs (Brew and Nagase, 2010), can reduce the damaging effects of MMPs on the kidney. Renal fibrosis, a common outcome of CKD, plays a pivotal role in the development of renal insufficiency (Liu, 2011). In animal models of kidney injury, such as unilateral urethral obstruction (UUO), the absence of TIMP3 has been found to activate multiple signalling pathways, including TGFβ/Smad, TACE/TNF-α, and mitogen-activated protein kinase pathways. This leads to heightened renal fibrosis, injury, and apoptosis following UUO (Wang et al., 2014). Another study also demonstrated that high expression of TIMP3 alleviates kidney damage through the TGFβ pathway (Chatterjee et al., 2023). In human kidney disease, TIMP3 is primarily upregulated in proximal renal tubules and vascular compartments to mitigate interstitial nephritis and fibrosis, potentially serving as a protective mechanism to minimize kidney damage (Kassiri et al., 2009). Therefore, the findings of this study align with previous research and suggest that an elevated level of TIMP3 has a safeguarding impact on CKD. As with psoriatic arthritis, pharmacogenomics may be useful in selecting etanercept for treatment (Murdaca et al., 2017). Some studies have shown that pracinostat increases the expression of TIMP3, which plays an important role in inhibiting disease progression in human glioma (Chen et al., 2022). This may provide valuable insights for the treatment of CKD.

Diabetic nephropathy is a key factor in the development of CKD (Molitch et al., 2003), with its defining features being an increase in albuminuria, decline in the glomerular filtration rate (GFR), and loss of podocytes. In a retrospective study, the potential roles of TIMP3 and its related noncoding RNA in the progression of diabetic nephropathy were investigated (Casagrande et al., 2021). An example of the moderation of podocellular damage in diabetic nephrotic disease is the overexpression of non-coding RNA (lincRNA) 4930556M19Rik which downregulates miR-27a-3p and upregulates TIMP3 (Fan and Zhang, 2020). Deficiencies in TIMP3 worsen diabetic renal injury, characterized by mesangial dilation and increased microalbuminuria, and affecting the progression of diabetic nephropathy through changes to Akt, ERK1/2, and PKC signaling pathways (Basu et al., 2012). Another study has confirmed that TIMP-3 deficiency contributes to the development of diabetic nephropathy through FoxO1/STAT1 interactions (Fiorentino et al., 2013). These studies have indicated that TIMP3 has potential as a biomarker or therapeutic target for renal diabetes (Casagrande et al., 2021). In this study, high levels of TIMP3 were found to increase eGRFcrea (No DM), but no causal relationship was identified for eGRFcrea (DM). Therefore, additional genetic research is needed to clarify the correlation between blood TIMP3 levels and kidney function in diabetic patients.

The current study has a number of advantages. First of all, it constitutes the initial MR study to examine the plausible impact of escalated TIMP3 levels in reducing renal harm and promoting eGFRcrea (No DM) in a particular population subset, thereby establishing a causal link. Secondly, to reduce the possibility of confounding effects and reverse causality, this MR study makes use of two enormous sets of GWAS data from European populations. Thirdly, kidney function is less susceptible to other factors, and the MR Analysis provides insight into the long-term effects of genetically determined TIMP3 levels on that risk.

There are limitations to this study that need to be acknowledged. Firstly, every individual we have researched is of European ancestry. Hence, further research is needed to ascertain whether the generalization of findings is possible for individuals of other ethnicities. Secondly, due to a lack of identified instrumental variables, we had to adopt a more lenient screening criterion (r2:0.1) to investigate the causal connection between TIMP-3 and renal function. This may potentially affect the reliability of our findings. Additionally, due to our reliance on pooled GWAS data and the absence of complete clinical subject information, we were unable to conduct a subgroup analysis to explore possible variations further.

## 5 Conclusion

Our study has shown that high levels of TIMP3 are linked with a lower risk of chronic kidney disease and an increased eGFRcrea (No DM). These results indicate a potential new method for preserving renal function.

## Data Availability

The datasets presented in this study can be found in online repositories. The names of the repository/repositories and accession number(s) can be found in the article/Supplementary material.
